# Incidence of acute kidney injury among high-risk adult patients undergoing iso-osmolar contrasted computed tomography scans: a prospective cohort study

**DOI:** 10.4314/ahs.v25i4.18

**Published:** 2025-12

**Authors:** Miriam Nakku, Zeridah Muyinda, Valeria Nabbosa, Aloysius G Mubuuke, Joseph B Baluku, Deborah Babirye, Jonathan Walubembe, Diana O Angom, Kevina Nalwoga, Robert Kalyesubula

**Affiliations:** 1 Department of Radiology, School of Medicine, College of Health Sciences, Makerere University, Uganda; 2 Department of Radiology, Mulago National Referral Hospital, Uganda; 3 Department of Radiology, Uganda Cancer Institute; 4 Department of Pulmonology, Kiruddu National Referral Hospital, Uganda; 5 Department of Internal Medicine, Mulago National Referral Hospital, Uganda

**Keywords:** Acute kidney injury, iso-osmolar, tomography, cohort study

## Abstract

**Background:**

The safety of iso-osmolar contrast media (IOCM) in patients at risk of contrast-induced kidney injury (CIAKI) is not well-established.

**Objective:**

To determine the incidence of CIAKI among high-risk adult patients undergoing iso-osmolar contrasted Computed Tomography (CT) scans.

**Methods:**

A prospective descriptive cohort study of patients at high risk of CIAKI was done. Questionnaires were used to collect clinical and examination findings. Blood samples were collected at baseline and at 48 hours post-administration of IOCM to assess changes in serum creatinine. Those found to have a ≥ 0.5mg/dl absolute increase in serum creatinine from the baseline within 48 hours of contrast administration were considered to have developed CIAKI. Pearson's Chi-square test was used to compare patients with and without CIAKI. Logistic regression models were used to determine associations of CIAKI.

**Results:**

The cumulative incidence of CIAKI was 9% (20/223), (95%CI – 5.8451-13.5215). No factors were independently associated with CIAKI in this study.

**Conclusion:**

The incidence of CIAKI among high-risk adult patients undergoing CT scans was 9%. There were no factors significantly associated with CIAKI.

## Background

The use of iodinated contrast media (CM) has increased globally^1^. This is because CM allows excellent visualization of structures, enabling clinicians to make proper diagnoses; improving patient management and quality of life^2^.

Despite the improved quality of CM, many adverse reactions like acute kidney injury (AKI) remain a major concern for clinicians^3^,^4^. These reactions may be mild, moderate or severe and can occur immediately or later post contrast administration^1^,^5^. Most of these adverse effects can be minimized or treated early when anticipated^6^.

The incidence of adverse effects is reported to be significantly reduced with the use of iso-osmolar contrasted media (IOCM)^4^,^7^. According to international guidelines, both iso-osmolar and low-osmolar CM are recommended for use in patients with increased risk of contrast-induced acute kidney injury (CIAKI)^3^,^8^-^10^.

Patients with co-morbidities like cancer, diabetes mellitus (DM) and renal impairment have a higher risk of acquiring adverse effects^11^,^12^. Therefore, care must be taken to prevent disease progression, reduced quality of life, increased hospital stay or even death by using appropriate CM^2^,^13^-^16^.

The incidence of CIAKI is reported to be as high as 50% in high-risk patients with majority of studies reporting ranges between 20-30%^17^. This has been attributed to the volumes and type of contrast media used. With the use of IOCM, the incidence is reported to be as low as 6.3%^12^ making it a safer contrast agent^17^,^18^.

The purpose of this study was to determine the incidence of acute kidney injury among high-risk adult patients undergoing iso-osmolar contrasted computed tomography (CT) scans.

This will provide a scientific guide on the safety profile of IOCM use among high-risk patients, allowing timely imaging, diagnosis and management, improving quality of life.

## Materials and methods

### Study aim, design, population and setting

This was a prospective descriptive cohort study aimed at determining the incidence of AKI among high-risk adult patients undergoing iso-osmolar contrasted CT scans at three centers in Kampala, Uganda. It was conducted at the Uganda Cancer Institute (UCI), St Francis Hospital Nsambya (SFHN) and Mulago National Referral Hospital (MNRH): all of which are tertiary institutions receiving patients from all over the country. The study population consisted of all consenting high-risk adult patients (chronic kidney disease (CKD), DM, cancer, hypertension, Human Immuno-deficiency Virus (HIV) or multiple co-morbidities) that presented for a contrasted CT scan.

**Figure 1:**
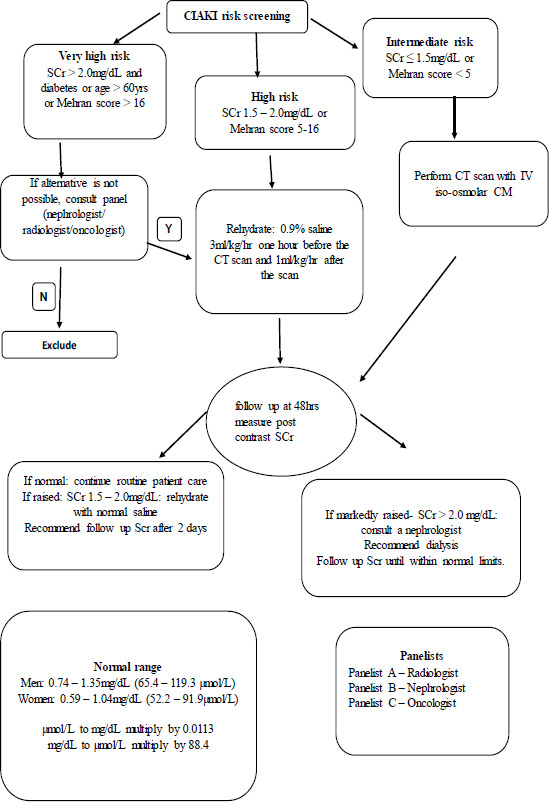
Protocol for contrasted CT risk according to American College of Radiology (ACR) criteria with modification. Goldfarb et al, 2009

A protocol for the purpose of renal protection (see figure 1 below) was developed by panelists including a radiologist, nephrologist and an oncologist prior to commencement of the study that was followed.

We excluded very ill patients whose vitals could not be taken or who could not be well positioned for the CT scan, patients who had had a contrasted CT scan performed in the previous three weeks and those with a baseline serum creatinine (SCr) of > 2.0mg/dL that were considered unfit by the panel.

### Data collection

Consecutive sampling was used. The independent variables were: age, sex, place of residence, occupation, level of education, marital status, type CT scan type, volume of CM, comorbidities like CKD, hypertension, DM, cancer, HIV, concurrent drug use and other therapies (Non-Steroidal Anti-Inflammatory Drugs, Highly Active Antiretroviral Therapy, chemotherapy, radiotherapy, oral anti-hyperglycemic agents, and anti-hypertension medication). The dependent variables were: CIAKI and adverse effects.

SCr levels were measured at baseline and at 48hours post contrasted CT scan using IOCM [Visipaque - iodixanol, General Electric Company (GE Healthcare)]. Patients found with an absolute increase of SCr ≥ 0.5 mg/dl from the baseline were considered to have developed CIAKI.

### Data analysis

Data was electronically entered using Microsoft Access 2016, exported into Excel 2016 and analyzed using STATA version 17.0

Statistical analysis was done in 3 stages. In the univariate analysis, data was presented as frequencies, proportions, means and medians with their measures of dispersion. The cumulative incidence of CIAKI was measured as the proportion of patients that developed CIAKI at 48 hours.

At the bivariate stage, a Chi-square test was used to measure association between the dependent and independent variables. Independent factors with a Chi-square p-value ≤ 0.2 were considered to have a significant association with development of CIAKI and were further tested using logistic regression to check for the association with usage at the multivariate stage.

## Results

223 participants were enrolled. The mean age was 56.5 (SD 14.5) years. 45.3% had a BMI of 18.5-24.9, 16.1% below 18.5, 23.8% between 25-29.9 and 14.8% above 30.

### Cumulative incidence of CIAKI

The proportion of high-risk patients that developed CIAKI was 9% (20) (95% CI 5.8451-13.5215) out of the 223 participants. Characteristics of participants are shown in tables 2-3. The statistically significant factors (p < 0.2) among participants that developed CIAKI were age, diabetes, cancer, metformin use, reduced urine output, Mehran risk score and adverse effects: pain at injection site, nausea and vomiting.

**Table 2:** Presence of chronic Illness, type of CT scan and Development of CIAKI

		Total n (%)	Developed n (%)	CIAKI	P-value
**Type of Computed Tomography scan exam**			**No**	**Yes**	
	Head	28(12.6)	26(92.9)	2(7.1)	0.925
	Chest	66(29.6)	60(90.9)	6(9.1)	
	Abdomen	109(48.9)	100(91.7)	9(8.3)	
	Pelvic	1(0.5)	1(100)	0(0)	
	Angiogram	7(3.1)	6(85.7)	1(14.3)	
	Others	12(5.4)	10(83.3)	2(16.7)	
**Chronic Kidney Disease**					
	No	219(98.2)	200(91.3)	19(8.7)	0.257
	Yes	4(1.8)	3(75)	1(25)	
**Diabetes**					
	No	181(81.2)	167(92.3)	14(7.7)	**0.181**
	Yes	42(18.8)	36(85.7)	6(14.3)	
**Hypertension**					
	No	116(52.0)	106(91.4)	10(8.6)	0.850
	Yes	107(48.0)	97(90.7)	10(9.3)	
**HIV**					
	No	150(67.3)	137(91.3)	13(8.7)	0.821
	Yes	73(32.7)	66(90.4)	7(9.6)	
**Cancer**					
	No	24(10.8)	20(83.3)	4(16.7)	**0.162**
	Yes	199(89.2)	183(92.0)	16(8.0)	
**Heart Disease**					
	No	222(99.5)	202(91.0)	20(9.0)	0.753
	Yes	1(0.5)	1(100)	0(0)	
**Liver Disease**					
	No	212(95.1)	192(90.6)	20(9.4)	0.286
	Yes	11(4.9)	11(100)	0(0)	

**Table 3:** History of allergies, eGFR, adverse effects and development of CIAKI

		Total n (%)	Developed	CIAKI n (%)	P-value
**History of allergies**			**No**	**Yes**	
	Yes	16(7.2)	14(87.5)	2(12.5)	0.608
	No	207(92.8)	189(91.3)	18(8.7)	
**eGFR**					
	<60 mL/min/1.73m^2^	28(12.6)	25(89.3)	3(10.7)	0.730
	≥60 mL/min/1.73m^2^				
		195(87.4)	178(91.3)	17(8.7)	
**Hematocrit Levels**					
	Above 35 – Safer	176(78.9)	162(92.1)	14(7.9)	0.305
	Below 36 – At Risk	47(21.1)	41(87.2)	6(12.8)	
**Volume of CM**					
	Below 60ml – Safer	89(39.9)	82(92.1)	7(7.9)	0.638
	Above 60ml – At Risk	134(60.1)	121(90.3)	13(9.7)	
**Mehran Risk Score**					
	Less or Equal to 5	172(77.1)	160(93)	12(7)	**0.097**
	6 – 10	43(19.3)	37(86.1)	6(13.9)	
	11 – 16	8(3.6)	6(75)	2(25)	
**Other adverse effects**					
	Yes	156(70.0)	137(87.8)	19(12.2)	**0.010**
	No	67(30.0)	66(98.5)	1(1.5)	
**Severity (Onset)**					
**Pain at injection site**					
	No Reaction	80(35.9)	77(96.3)	3(3.7)	**0.041**
	Mild – No Treatment	143(64.1)	126(88.1)	17(11.9)	
**Flushing**					
	No Reaction	220(98.6)	200(90.9)	20(9.1)	0.584
	Mild – No Treatment	3(1.4)	3(100)	0(0)	
**Dizziness**					
	No Reaction	190(85.2)	174(91.6)	16(8.4)	0.492
	Mild – No Treatment	33(14.8)	29(87.9)	4(12.1)	
**Nausea**					
	No Reaction	173(77.6)	162(93.6)	11(6.4)	**0.011**
	Mild – No Treatment	50(22.4)	41(82)	9(18)	
**Vomiting**					
	No Reaction	208(93.3)	192(92.3)	16(7.7)	**0.013**
	Mild – No Treatment	15(6.7)	11(73.3)	4(26.7)	
**Headache**					
	No Reaction	188(84.3)	172(91.5)	16(8.5)	0.790
	Mild – No Treatment	34(15.3)	30(88.2)	4(11.8)	
	Moderate – Treatment Necessary	1(0.4)	1(100)	0(0)	
**Urticaria (skin rash)**					
	No Reaction	221(99.1)	201(90.9)	20(9.1)	0.656
	Mild – No Treatment	2(0.9)	2(100)	0(0)	
**Abdominal Cramps**					
	No Reaction	219(98.2)	199(90.9)	20(9.1)	0.818
	Mild - No Treatment	3(1.4)	3(100)	0(0)	
	Moderate – Treatment Necessary	1(0.4)	1(100)	0(0)	
**Itching**					
	No Reaction	216(96.9)	197(91.2)	19(8.8)	0.617
	Mild – No Treatment	7(3.1)	6(85.7)	1(14.3)	
**History of reduced urine output**					
**urine output**					
	Yes	20(9.0)	16(80)	4(20)	0.070
	No	203(91.0)	187(92.1)	16(7.9)	

### Factors associated with development of CIAKI

As shown in table 4, there were no independent factors statistically significantly associated with CIAKI.

**Table 4:** Best predictors for development of CIAKI Multivariate Analysis for factors associated with CIAKI

Outcome: Developed CIAKI	Adjusted Odds Ratios (95% CI)	P>z
**Age of Patient**		
Below 60 Years	1.00	
Above 60 Years	1.17(0.34-3.98)	0.798
**Chronic Illness: Diabetes**		
No	1.00	
Yes	0.94(0.26-3.41)	0.935
**Chronic Illness: Cancer**		
No	1.00	
Yes	0.3(0.06-1.5)	0.143
**History of reduced Urine Output**		
Yes	1.00	
No	2.02(0.47-8.69)	0.342
**Mehran Risk Score**		
11 – 16	1.00	
Less than or Equal 5	0.29(0.03-2.88)	0.295
6 – 10	0.44(0.05-3.87)	0.463
**Adverse Effects**		
No	1.00	
Yes	10.43(0.79-136.64)	0.074
**Onset Severity: Pain**		
No Reaction	1.00	
Mild – No Treatment	1.01(0.19-5.16)	0.989
**Onset Severity: Nausea**		
No Reaction	1.00	
Mild – No Treatment	1.87(0.63-5.55)	0.256
**Onset Severity: Vomiting**		
No Reaction	1.00	
Mild – No Treatment	1.5(0.31-7.29)	0.610

## Discussion

The cumulative incidence of CIAKI was 9%. This is similar to many studies in high-risk patients with IOCM use where incidences of < 12% have been noted^3^,^10^,^19^. This is because IOCM is safer for use in high-risk patients compared to other CM agents where incidences as high as 20-30% have been reported^10^,^17^,^20^,^21^. The safety of IOCM has been attributed to its reduced cytotoxicity and nephrotoxicity^19^.

In a similar study carried out in Germany by Werner et al an incidence of 6.3% was registered. This study had a slightly higher incidence possibly because higher volumes of CM > 60mls were used compared to those (60mls) used in Germany^12^. The use of low doses of CM reduces the risk of CIAKI.

In another study conducted in Kenya by Kwasa et al using IOCM in patients with inflammation, the incidence of CIAKI was found to be 9.92%. Inflammation is however present in most of the risk factors predisposing to CIAKI and therefore these results were comparable to those in this study^22^.

In this study, there were no factors independently associated with development of CIAKI at the multivariate level. This could be due to the different ethnic population in which the study was carried out and the fact that IOCM is regarded safe among the high-risk groups^3^,^10^,^17^,^19^,^20^. Furthermore, the use of the reno-protective protocol developed in this study could have played a role.

Therefore, this study demonstrated that IOCM can be safely used among high-risk patients with minimal fear of developing CIAKI. The strength of the study lies in its sizable sample size representative of the high-risk patients drawn from three different centers. The findings generated from the study thus contribute to literature on the incidence of developing acute kidney injury following IOCM use among high-risk patients.

Limitation of the study was underestimating the incidence of CIAKI given the short duration of follow up. Despite the limitation, the findings are useful to guide on CM use in imaging high-risk patients who have always been approached with fear. We do recommend further research specifically looking at a longer follow up period.

## Conclusion

The incidence of CIAKI among high-risk adult patients undergoing iso-osmolar contrasted CT scans in this study was 9%, which is relatively low compared to previously reported literature. IOCM can therefore be used among high-risk adult patients during their clinical management.

## Figures and Tables

**Table 1:** Social Demographics of patients enrolled

**Sex**		
	Male	106(47.5)
	Female	117(52.5)
**Age (categorized)**		
	25 and Below	3(1.4)
	26 – 45	49(21.9)
	46 – 65	104(46.6)
	66 – 85	64(28.7)
	86 and Above	3(1.4)
**Occupation**		
	Farmer	88(39.5)
	Teacher	11(4.9)
	Business Man/Woman	62(27.8)
	Health Worker	8(3.6)
	Others	54(24.2)
**Education**		
	Primary	118(52.9)
	Secondary	47(21.1)
	Tertiary	58(26.0)
**Marital Status**		
	Single	43(19.3)
	Married	180(80.7)
**BMI**		
	Under Weight	36(16.1)
	Normal Weight	101(45.3)
	Overweight	53(23.8)
	Obese	33(14.8)

## Data Availability

The datasets analyzed in this study are available from the corresponding author on reasonable request.
